# Integrated Use of *Aureobasidium pullulans* Strain CG163 and Acibenzolar-S-Methyl for Management of Bacterial Canker in Kiwifruit

**DOI:** 10.3390/plants8080287

**Published:** 2019-08-15

**Authors:** Huub de Jong, Tony Reglinski, Philip A.G. Elmer, Kirstin Wurms, Joel L. Vanneste, Lindy F. Guo, Maryam Alavi

**Affiliations:** 1 The New Zealand Institute for Plant & Food Research Limited, Hamilton 3214, New Zealand; 2 School of Biological sciences, Faculty of Science, Auckland University, Auckland 1010, New Zealand; 3 The New Zealand Institute for Plant & Food Research Limited, Mt. Albert Research Centre, Sandringham 1142, New Zealand

**Keywords:** biocontrol, induced resistance, *Aureobasidium pullulans*, *Pseudomonas syringae* pv. *actinidiae*, *Actinidia chinensis*, acibenzolar-S-methyl, kiwifruit defence, priming

## Abstract

An isolate of *Aureobasidium pullulans* (strain = CG163) and the plant defence elicitor acibenzolar-S-methyl (ASM) were investigated for their ability to control leaf spot in kiwifruit caused by *Pseudomonas* *syringae* pv. *actinidiae* biovar 3 (Psa). Clonal *Actinidia chinensis* var. *deliciosa* plantlets (‘Hayward’) were treated with ASM, CG163 or ASM + CG163 at seven and one day before inoculation with Psa. ASM (0.2 g/L) was applied either as a root or foliar treatments and CG163 was applied as a foliar spray containing 2 × 10^7^ CFU/mL. Leaf spot incidence was significantly reduced by all treatments compared with the control. The combination of ASM + CG163 had greater efficacy (75%) than either ASM (55%) or CG163 (40%) alone. Moreover, treatment efficacy correlated positively with the expression of defence-related genes: pathogenesis-related protein 1 (PR1), β-1,3-glucosidase, Glucan endo 1,3-β-glucosidase (Gluc_PrimerH) and Class IV chitinase (ClassIV_Chit), with greater gene upregulation in plants treated with ASM + CG163 than by the individual treatments. Pathogen population studies indicated that CG163 had significant suppressive activity against epiphytic populations of Psa. Endophytic populations were reduced by ASM + CG163 but not by the individual treatments, and by 96–144 h after inoculation were significantly lower than the control. Together these data suggest that ASM + CG163 have complementary modes of action that contribute to greater control of leaf spotting than either treatment alone.

## 1. Introduction

*Pseudomonas syringae* pv. *actinidiae* (Psa) is the causal agent of bacterial canker in kiwifruit (*Actinidia* spp.). Psa infections of kiwifruit have been caused by five related, but genetically distinct lineages of the pathogen. Biovar 1 was first isolated in Japan (1984) and Italy (1992), while biovar 2 was isolated in Korea (1997). The global production of kiwifruit was not greatly affected by those early occurrences or by more recent biovars (5 and 6) isolated in Japan in 2012 and 2015 [1]. However, the outbreak of Psa in Italy in 2008 caused by biovar 3 (Psa), was later found in other major kiwifruit-growing regions in France [2], Chile and New Zealand [3,4], causing a destructive disease and presenting a major threat to the global kiwifruit industry [5]. Psa3 was first discovered in New Zealand on November 2010 on the yellow-fleshed *A. chinensis* Planch. var. *chinensis* Planch ‘Hort 16A’ and subsequently on the green fleshed ‘Hayward’ *A. chinensis* var *deliciosa*, and by 2018, it had spread to 92% of kiwifruit orchards [6]. The rapid spread in New Zealand may be attributed to the virulence of Psa, a relatively low genetic base (only three commercial cultivars), and the scarcity of products which were available for the initial control of Psa [7], as well as kiwifruit being the dominant crop by far in a region where climatic conditions favour infection almost year round [1]. The economic impact of the Psa outbreak in New Zealand resulted in a decision by the industry to remove the highly susceptible ‘Hort16A’ and replacement with a more tolerant cultivar (‘Gold3’). Infection in the ‘Hayward’ cultivar was generally less severe, with bud browning and leaf spotting in infected vines. It has been estimated that the cost of lost exports was as high as NZ$930 million by 2014 [1].

Psa is currently managed through the use of chemical control products, as well as cultural control through the regular removal of infected and dead canes. The main products used for control of Psa can be divided into three broad groups: copper products, bactericides (Kasumin^®^, which contains kasugamycin) and a plant defence elicitor (Actigard^®^, containing acibenzolar-S-methyl, ASM). Copper-based products come in the form of: copper oxide and copper hydroxide [8]. However, the use of copper is generally not regarded as sustainable, because of either restrictions on their use (limiting the number of sprays per season), environmental concern such as heavy metal accumulation in soils [9] or the development of pathogen resistance to copper. Copper-resistant Psa strains have already been isolated in New Zealand, and resistance could be acquired by other Psa populations through horizontal gene transfer [10]. Even though no reports have stated a loss in disease control efficacy in the field, the industry recognizes the need to move to integrated pest management for Psa, and this includes moving away from less desirable products such as copper and bactericides while maintaining control of Psa.

There is increasing interest in the use of plant defence elicitors like ASM for sustainable plant disease control. ASM is sold commercially under the names of Bion^®^ or Actigard and has been reported to protect plants against a broad spectrum of plant pathogens [11,12,13]. ASM has no direct anti-microbial activity per se but instead operates as a functional analogue of salicylic acid (SA) [14] leading to the upregulation of pathogenesis-related (PR) genes and an increase in resistance to subsequent pathogen attack. The expression of PR1 has been used as a marker to monitor defence activation by ASM in several plant species, including kiwifruit [12]. ASM was shown to demonstrate efficacy against Psa in glasshouse trials [7,15] and was introduced into the Psa management programme in New Zealand kiwifruit orchards in 2011. However, applications of ASM are limited to a maximum of four per season, with none allowed between flowering and harvest, to prevent residues in the fruit [8], and alternative approaches are sought to broaden control options for growers. 

Biocontrol agents (BCAs) may offer an environmentally attractive option for Psa management. Non-bacterial BCAs such as *Aureobasidium pullulans* have been registered for their use in pome fruit against fire blight in Europe (Blossom Protect^®^) [16]. In greenhouse studies in Italy, Blossom Protect reduced leaf infection caused by Psa [17]. Treatment of avocado fruit with *A. pullulans* resulted in an increase in chitinase and β-1,3-glucosidase activity, suggesting that these BCAs also have the potential to enhance host defences [18]. Previous studies have also established the use of BCAs in combination with ASM for enhanced protection against plant pathogens. Combinations of ASM and plant growth-promoting rhizobacteria (PGPR) were shown to have an additive effect on disease suppression against bacterial wilt (*Ralstonia solanacearum*), bacterial speck (*P. syringae* pv. *tomato*) and fusarium crown and root rot (*Fusarium oxysporum* f. sp. *radices-lycopersici*) in tomato [19,20,21]. In addition, greater disease suppression and lower variability against *Fusarium oxysporum* and *Pythium debaryanum* was achieved when *Trichoderma hamatum*, *Paecilomyces lilacinus*, SA and ASM were combined [22]. 

In kiwifruit, a study found that using multiple endophytic strains of *Pseudomonas* sp. isolated from manuka (*Leptospermum scoparium*) were more effective against Psa wound colonisation in kiwifruit plants when used in combinations, than when used individually [23]. The additive effect of combining multiple antagonistic bacteria was speculated to be contributing to a wider range of biocontrol activity against Psa.

In this study, we investigated the use of an *A. pullulans* strain (strain CG163) alone, and in combination with ASM to control Psa in kiwifruit. This isolate of *A. pullulans* is based on a commercial formulation (Aureo^®^Gold; https://www.etec.co.nz/product-page/aureo-gold) and was recently developed for control of Psa in kiwifruit (International Publication Number: WO2018047123). Field efficacy of AureoGold was demonstrated over a wide range of Psa disease pressure conditions and ranged from 40% to 60% disease control. 

Our hypothesis is that the complementary activities of CG163 and ASM may offer a more effective and sustainable control of Psa when combined, than when used individually.

## 2. Results

### 2.1. Disease Incidence on Plants Treated with CG163 or ASM, Alone or in Combination

**Figure 1:**
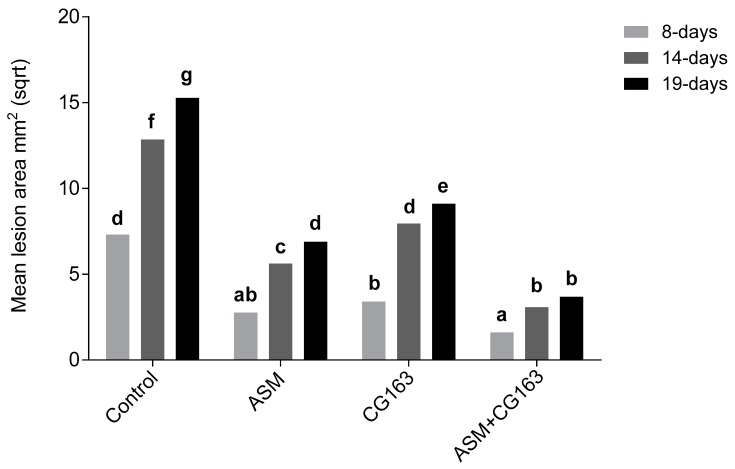
Mean lesion area in *Actinidia chinensis* var. *deliciosa* ‘Hayward’ potted plants treated with acibenzolar-S-methyl (ASM), *Aureobasidium pullulans* isolate CG163 (CG163), combination of ASM and CG163 (ASM + CG163) or untreated (control), at seven and one days before inoculation with 2 × 10^7^ CFU/mL *Pseudomonas syringae* pv. *actinidiae* (Psa). Disease symptoms were assessed 8, 14 and 19 days post inoculation. Values are means of 15 biological replicates with six inoculation spots on three leaves per rep. Plant replicate was included as a block effect for statistical analysis. Bars with different letters, based on square-root-transformed means are significantly different according to Fisher’s Least Significant Difference test (LSD) at the 5% level.

By eight days post inoculation, the lesions on all the treated plants were significantly smaller (F_3,56_ = 52.13, *p* < 0.001) than those on the untreated control plants (Figure 1). By nineteen days post inoculation, the average lesion size on plants treated with ASM in combination with CG163 (~20 mm^2^) was significantly smaller (F_6,112_ = 23.9, *p* < 0.001) than that on plants that received any of the other treatments. The reduction in the area of leaf necrosis on plants treated with ASM and CG163 at 19 days post inoculation was 75% compared with 55% and 40% on plants treated with only ASM or CG163, respectively.

### 2.2. Effect of Timing of CG163 and ASM Applications on Disease Incidence

**Figure 2:**
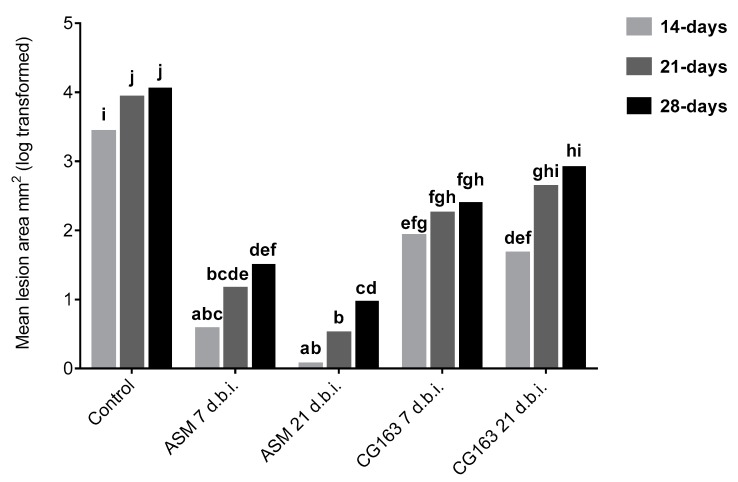
Mean lesion area in *Actinidia chinensis* var. *deliciosa* ‘Hayward’ potted plants treated with acibenzolar-S-methyl (ASM), *Aureobasidium pullulans* strain CG163 (CG163) or untreated (control) 21 or 7 days before inoculation (d.b.i.) with 2 × 10^7^ CFU/mL *Pseudomonas syringae* pv. *actinidiae*. Disease symptoms were assessed 14, 21 and 28-days post inoculation. Values are means of 12 biological replicates with six inoculation spots on three leaves. Each biological replicate consisted of an average of six inoculation spots on three leaves per rep. Plant replicate was included as a block effect for statistical analysis. Bars with different letters, based on log-transformed means are significantly different according to Fisher’s Least Significant Difference test (LSD) at the 5% level.

Plants treated with ASM or CG163 treatments applied 21 or 7 days before inoculation had significantly (F_4,54_ = 15.81, *p* < 0.001) smaller lesions at each individual assessment date than the untreated control (Figure 2).

### 2.3. Persistence of A. pullulans CG163 on A. Chinensis var. Deliciosa ‘Hayward’ Leaves

**Figure 3:**
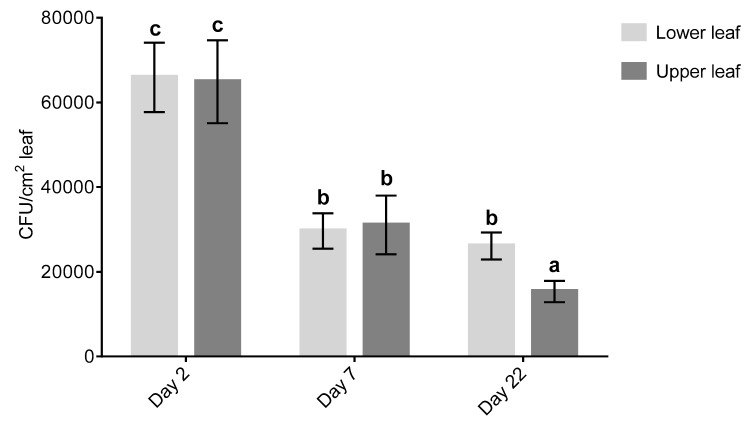
Average populations of *Aureobasidium pullulans* CG163 (CG163) (CFU/cm^2^ leaf) on upper immature still expanding and lower fully expanded *Actinidia chinensis* var. *deliciosa* ‘Hayward’ leaves. Five leaf discs per plant were taken 2, 7 and 22 days after CG163 spray treatment. Values are means ± standard errors of nine biological replicates. Each biological replicate consisted of an average of five leaf discs per leaf. Different letters are based on back-transformed average CG163 populations and are significantly different according to Fisher’s Least Significant Difference test (LSD) at the 5% level.

Populations of CG163 declined significantly (F_5,48_ = 11.09, *p* < 0.001) from approximately 6.5 × 10^4^ at 2 days after treatment to 3 × 10^4^ CFU/cm^2^ at 7 days after treatment on upper and lower leaves (Figure 3). The CG163 population on lower fully expanded leaves remained stable between 7 and 22 days after treatment, whereas those on upper immature leaves showed a significant (F_5,48_ = 11.09, *p* < 0.001) decline over the same period.

### 2.4. Effects of ASM and A. pullulans CG163 Treatments on Plant Growth

**Figure 4:**
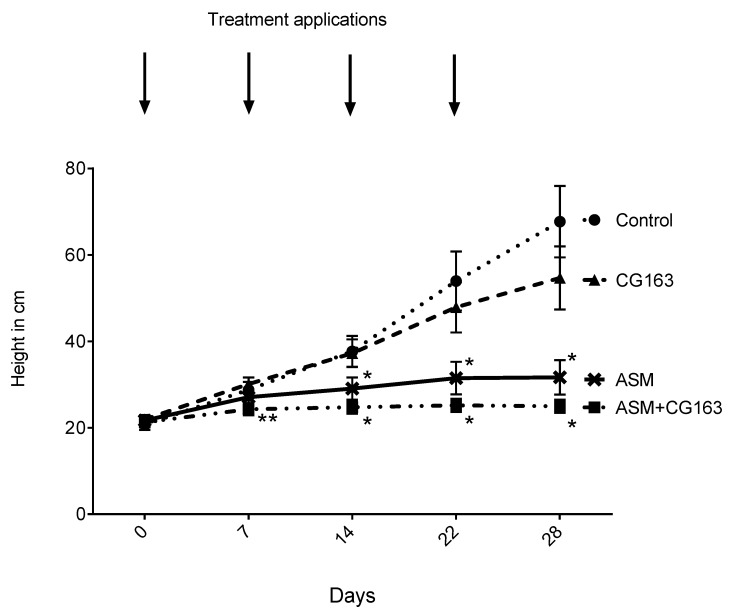
Plant growth of *Actinidia chinensis* var. *deliciosa* ‘Hayward’ potted plants treated with acibenzolar-S-methyl (ASM), *Aureobasidium pullulans* strain CG163 (CG163), combination of ASM and CG163 (ASM + CG163) or untreated (control). Weekly application of the treatments was started at T = 0 days and continued until T = 22 days. Weekly measurements of plant height were taken, measuring from the bottom of the stem near the soil to the tip of the stem. Values are means ± standard errors of 15 biological replicates. Asterisks indicate statistically significant differences between the different treatments based on back-transformed average plant length (**: ASM + CG163 statistically different from CG163, *: ASM and ASM + CG163 both statistically different from CG163 and untreated control) according to Fisher’s Least Significant Difference test (LSD) at the 5% level.

Repeat applications of ASM resulted in retardation of plant growth (Figure 4). After two weeks, plants treated with ASM and ASM + CG163 at day 0 and day 7 were significantly (F_3,56_ = 7.1188, *p* < 0.001) shorter than the untreated plants or the plants treated with CG163 alone (Figure 4). Moreover, no, height increase was recorded for plants treated with ASM or ASM in combination with CG163 treatments over the remainder of the experiment. In contrast CG163 alone did not significantly affect plant height, although there was an indication of slower growth observed compared with that in the control, starting from three weeks after the first treatment.

### 2.5. Treatment Effects on Psa Population

**Figure 5:**
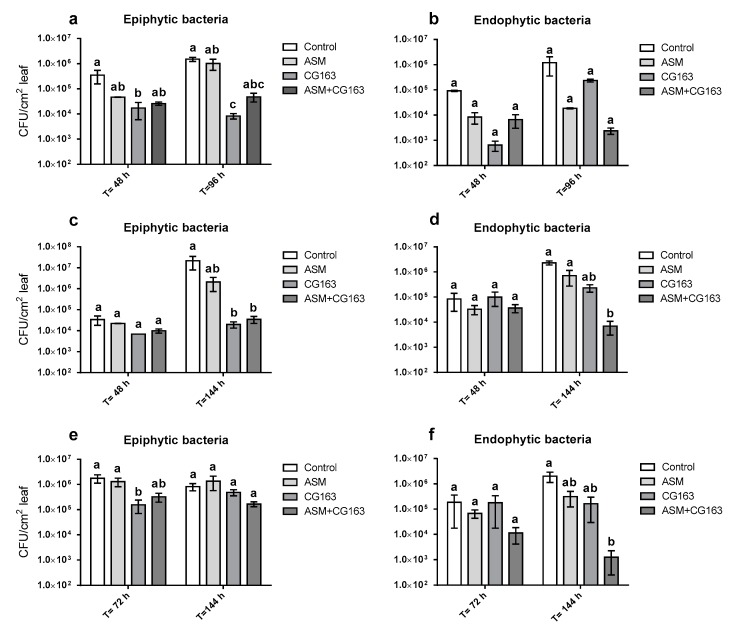
Three separate experiments, with time-points of 48 and 96 h (Experiment 1: (**a**,**b**)), 48 and 144 h (Experiment 2: (**c**,**d**)) and 72 and 144 h (Experiment 3: **e**,**f**), measuring average epiphytic (**a**,**c**,**e**) and endophytic populations (**b**,**d**,**f**) of *Pseudomonas syringae* pv. *actinidiae* (CFU/cm^2^ of leaf) in *Actinidia chinensis* var. *deliciosa* ‘Hayward’ potted plants. Plants were treated with acibenzolar-S-methyl (ASM), *Aureobasidium pullulans* isolate CG163 (CG163), combination of ASM and CG163 (ASM + CG163) or untreated (control) 7 and 1 day before inoculation with *Pseudomonas syringae* pv. *actinidiae*. Values are means ±SE of two biological replicates for Experiments 1 + 2 and six biological replicates for Experiment 3. Each biological replicate consisted of an average of one inoculation spot on three (Experiment 1 + 2) or two (Experiment 3) different leaves on the same plant. Bacterial populations just after inoculation consisted of two (Experiments 1 + 2) or six (Experiment 3) untreated biological replicates for all treatments, with each biological replicate consisting of an average of one inoculation spot on three (Experiments 1 + 2) or two (Experiment 3) different leaves on the same plant. Average epiphytic and endophytic bacterial populations were notated with different letters, signifying differences according to average back-transformed bacterial populations of a generalized linear mixed model (GLMM) with a 95% confidence interval.

Three experiments were performed to compare the effects of ASM and CG163 on epiphytic and endophytic Psa populations (Figure 5, Table S2). The epiphytic Psa population dropped from the initial inoculation concentration to approximately 1 × 10^2^ CFU/cm^2^ leaf in experiment 1, 1 × 10^3^ CFU/cm^2^ in experiment 2 and 2 × 10^2^ CFU/cm^2^ in experiment 3 immediately after inoculation. 

In all three experiments, there was a significant treatment effect on epiphytic populations. In experiment 1, the epiphytic population of Psa was ~120-fold lower (F_3, 42.8_ = 7.73, *p* < 0.001) at 96 h post inoculation, on CG163-treated plants than on ASM-treated or the untreated control (Figure 5a). In experiment 2, plants treated with CG163 and ASM + CG163 had epiphytic populations that were ~600-fold lower (F_3,14_ = 5.24, *p* < 0.012) than on the control at 144 h post inoculation (Figure 5c) and in experiment 3 CG163-treated plants had epiphytic population that were ~10-fold lower (F_3,29.3_ = 4.96, *p* < 0.005) than the control at 72 h post inoculation. No significant difference was found in epiphytic populations between the different treatments at 144 h post inoculation in experiment 3 (Figure 5e). 

Endophytic populations of Psa were recovered (200 CFU/cm^2^) immediately after inoculation in experiments 2 and 3 but not in experiment 1. ASM + CG163 was the only treatment which significantly reduced endophytic populations at 96 h post inoculation (Figure 5b), with a ~500-fold reduction (F_3,39.4_ = 3.59 *p* < 0.022) in endophytic population compared with that on the untreated control. There was no significant time x treatment interaction for endophytic populations.

### 2.6. Gene Expression Analysis of Plant Defence Responses by Real-Time Quantitative PCR (qPCR)

**Figure 6:**
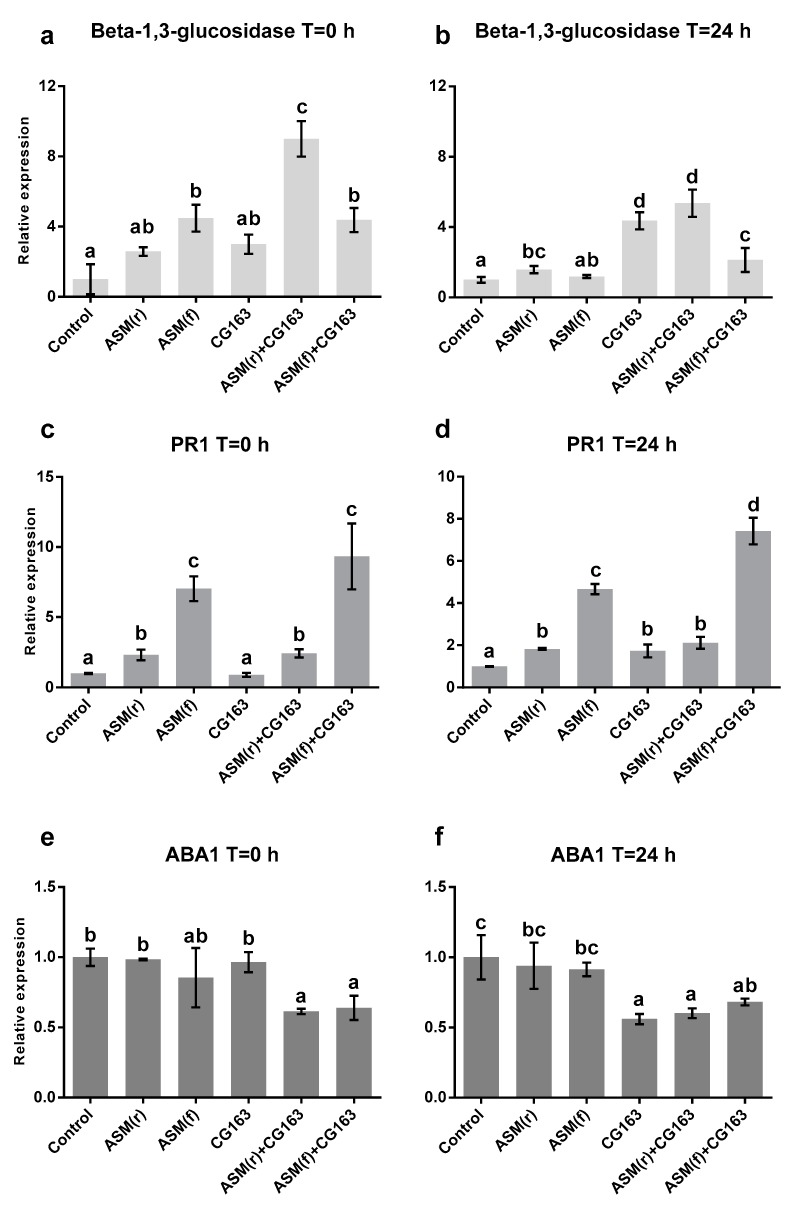
Relative expression of β-1,3-glucosidase (**a**,**b**), pathogenesis-related protein 1 (PR1) (**c**,**d**) and abscisic acid deficient 1 (ABA1) (**e**,**f**) genes in *Actinidia chinensis* var. *deliciosa* ‘Hayward’ potted plants treated with acibenzolar-S-methyl (ASM) root (r) or foliar-spray (f), *Aureobasidium pullulans* isolate CG163 (CG163), combination of ASM (r or f) and CG163 (ASM + CG163) or untreated (control) seven and one day before inoculation with 2 × 10^7^ CFU/mL *Pseudomonas syringae* pv. *actinidiae*. Relative expression was normalized against 40 s ribosomal protein gene and the protein phosphatase 2A gene. The basal transcript level in untreated plants (baseline treatment) was used as a further reference point for all calculations and is referred to with the value 1. Values are means ± standard errors of three independent biological replicates. Each biological replicate consisted of a pooled leaf sample from two ‘Hayward’ tissue cultures. Molecular samples were taken just before (0 h) and 24 h after Psa inoculation. Bars with different letters are based on back-transformed average relative expression and are significantly different according to Fisher’s Least Significant Difference test (LSD) at the 5% level.

At T = 0, β-1,3-glucosidase was significantly (F_5,12_ = 14.03, *p* < 0.001) upregulated by foliar ASM treatment and by ASM + CG163 compared with that in the untreated control, and was greatest in the root ASM + CG163 treatment (Figure 6). By 24 h after inoculation with Psa, β-1,3-glucosidase expression was elevated in all treatments (F_5,12_ = 21.4, *p* < 0.01) except foliar ASM, compared with that in the untreated control, and was greatest in CG163 and ASM + CG163. Pathogenesis related protein 1 (PR1) expression was significantly (F_5,12_ = 36.62, *p* < 0.01) induced by ASM treatments and by ASM + CG163 application, compared with that in the untreated control plants at 0 h and 24 h after Psa inoculation (Figure 6). CG163 did not induce PR1 expression at T = 0, but did induce an enhanced expression of PR1 at 24 h post inoculation, PR1 expression at T = 24 h was greatest (F_5,12_ = 65.19, *p* < 0.01) in plants treated with ASM + CG163. Abscisic acid deficient 1 (ABA1) expression was significantly downregulated (F_5,12_ =3.93, *p* < 0.027), compared with that in the control, by ASM + CG163 at T = 0 (Figure 6) and by CG163 and ASM + CG163 at 24 h after inoculation (F_5,12_ = 4.81, *p* < 0.014).

### 2.7. Gene Expression Analysis of Plant Defence Responses by CodeSet^®^ Nanostring

A broader screen of potential defence marker genes associated with the induction of host defence genes by ASM and CG163 was investigated. ASM, and CG163 to a lesser extent, upregulated genes associated with the salicylic acid (SA) pathway within 5–48 h of Psa inoculation (Table 1). There was an additive effect of ASM + CG163 on the expression SA-marker genes; pathogenesis-related protein 1 (PR1_P31), glucan endo 1,3-β-glucosidase (Gluc_PrimerH) and Class IV chitinase (ClassIV_Chit) (Table 1). This pattern corresponded with the phenotypic expression of induced resistance to Psa (Figure 1) and corroborated with earlier gene expression analyses (Figure 6). Pathogenesis-related protein 1 (PR1_P32) (F_15,32_ = 40.097, *p* < 0.001), downy mildew resistant 6 (DRM6) (F_15,32_ = 125.81, *p* < 0.001) and thaumatin-like protein (TLP_TG4) (F_15,32_ = 30.046, *p* < 0.001) were induced to a greater degree by ASM specifically than by CG163 (Table 1). Two isoforms of the pathogenesis-related protein 1 (PR1) family, PR1_P31 and PR1_P32, exhibited differential responses to ASM and CG163, with CG163 inducing greater expression of PR1_P31 (F_15,32_ = 27.767, *p* < 0.001), and ASM greater expression of PR1_PR32 (F_15,32_ = 40.097, *p* < 0.001) (Table 1). 

Genes associated with the jasmonic acid (JA) and ethylene (C2H4) pathways were generally unresponsive or downregulated by the treatments (Table 2). Treatments with ASM + CG163 caused upregulation of AP2 ERF2 (F_15,32_ = 15.2, *p* < 0.001) at 24 h after inoculation. However, this increase in expression was quite transient. 

ASM had a greater effect on the expression of genes associated with recognition and reaction oxygen species (ROS) than CG163 (Table 2). However, an additive effect of ASM + CG163 on the expression of RBOHF (F_15,32_ = 16.615, *p* < 0.001) and OX2_Zn_finger (F_15,32_ = 14.153, *p* < 0.001) genes was observed at 96 h after inoculation.

## 3. Discussion

The goal of this study was to determine whether an elicitor and a biological control agent would give more effective control of Psa on kiwifruit when combined than when applied individually. Previous studies have shown combining treatments with different modes of action, such as elicitors and BCAs, can result in better and more consistent disease control [15,19,20,21,22]. The elicitor chosen for this study is ASM, which has been shown to reduce Psa incidence in kiwifruit [12,15]. In this study, combining root or foliar ASM treatments with foliar sprays of *A. pullulans* (CG163), resulted in greater disease control of Psa on potted kiwifruit plants than that obtained with either treatment alone. Disease control correlated with the induction of plant defence genes and with direct suppression of Psa3 populations on leaves. It is proposed that the enhanced efficacy of the combination is attributable to the additive effects of their complementary modes of action. 

Root applications of ASM has previously been shown to induce greater control of Psa leaf spot in kiwifruit than spray treatments [15]. To physically separate the ASM treatment from foliar treatment with CG163, ASM was applied as a root drench in most experiments. Root applications of ASM 7 and 1 day before inoculation, or as a single application up to 21 days before inoculation, significantly reduced severity of Psa leaf necrosis compared with that in the untreated control. Psa endophytic populations were lower on ASM-treated plants than on untreated controls; however, the difference was not statistically significant. ASM application directly induced the kiwifruit defence response, as evidenced by the increased expression of β-1,3-glucosidase and PR1 in kiwifruit leaves prior to pathogen inoculation. These are putative markers of induced resistance pathways and have previously been studied in Psa/kiwifruit studies [24]. The expression of these genes were also significantly greater in ASM-treated plants at 24 h after pathogen inoculation, compared with that in controls, thus consistent with priming of the host defence response. β-1,3-glucosidases are hydrolytic enzymes that can activate phytohormonal signals and antimicrobial secondary metabolites through the removal of glycosyl residues, and enable the plant to respond immediately to pathogen invasion and activate the SA-pathway [25]. PR1 is among the best characterized PR genes and is often used as a marker for the induction of SA-dependent defence pathways and systemic acquired resistance (SAR) in plants [26]. The increases of PR1 in ASM-treated plants are consisted with ASM operating as a SA mimic [14], as well as ASM elevating PR1 in kiwifruit (shown in previous studies [15]). The overexpression of inducible defences can result in reductions in plant growth because of defence growth trade-offs [27]. This may explain the reduction in plant growth observed in our study after plants were treated twice with ASM. However, in practice, ASM treatments are limited to 4 sprays per season and they are not applied at weekly intervals. This could explain why this phenomenon has never been observed in commercial kiwifruit orchards. 

A broader assessment of defence gene expression in kiwifruit plants showed that marker genes of the SA pathway were strongly upregulated by foliar spray with ASM. Downy mildew resistant 6 (DRM6), thaumatin-like protein (TLP_TG4) and PR1_P32 were significantly induced in ASM-treated plants 5–96 h after Psa inoculation compared with the untreated control. AcDRM6 is induced by SA/pathogen treatments and is a possible modulator of the SA response. Overexpression of DRM6 led to increased efficacy against *P. syringae* pv. *tomato* in Arabidopsis [28]. TLP_TG4 comes from the PR5 family of thaumatin-like and osmotin-like proteins. Constitutive levels of PR5 are typically absent in healthy plants and are induced exclusively in response to wounding or pathogen attack [29]. PR5 proteins have shown antimicrobial activity against a *P. syringae* pathovar of soybean [30]. Increased resistance to pathogens in transgenic plants with elevated PR5 expression has also been demonstrated [31]. PR1_P32 a specific isoform of PR1 was most strongly upregulated compared with that in the untreated control.

Foliar sprays of an isolate of *A. pullulans* (CG163) significantly reduced disease severity caused by Psa. Foliar applications of CG163 7 and 1 day before inoculation, and a single application up to 21 days before inoculation, were shown to significantly reduce Psa leaf spot symptoms compared with those in the untreated control. CG163 population densities on fully expanded leaves declined significantly between 2 and 7 days after inoculation but stabilized thereafter up to at least 22 days after application. On immature developing leaves, there was a decline in the population density by 22 days; however, this is likely to be a function of leaf expansion during the period between application and measurement. Long-term survival on the phylloplane is considered to be a highly desirable trait for a BCA, requiring fewer treatment applications, leading to lower costs. On apple leaves, *A. pullulans* strains have been reported to persist for several months on the phylloplane; the colonization of apple leaves by *A. pullulans* occurred primarily in the veins, and population growth was attributed to possible nutrient availability [32,33]. Similarly, preliminary studies to determine spatial distribution patterns of CG163 on kiwifruit leaves have shown that populations are greatest adjacent to veins on the underside of the leaves (data not shown). The ability of CG163 to provide protection against Psa for up to 21 days despite a drop in population density that suggests its longer-term efficacy is not solely attributable to direct interactions with Psa, and that other mode(s) of action, such as the induction of host defences, may also be involved. 

Besides direct antagonistic effects, *A. pullulans* has also been shown to induce plant defence genes in grapevine cuttings, inducing pathogenesis-related proteins such as β-1,3-glucanase (Gluc) and PR6, seven days after inoculation with *Diplodia seriata*. In this study, host defence genes in ‘Hayward’ potted plants treated with CG163 were induced after inoculation with Psa. CG163 significantly elevated β-1,3-glucosidase and PR1 expression at 24 h after inoculation, suggesting a capacity for CG163 to ‘prime’ or condition ‘Hayward’ leaves, and therefore enabling an enhanced defence response to subsequent Psa challenge [34]. Priming of plants by the leaf-colonizing yeast BCA *Pseudozyma churashimaensis* was shown in pepper seedlings, causing increases in upregulation of PR4 and PR5 transcription 6 h after *Xanthomonas axonopodis* pv. *vesicatoria* inoculation [35]. However, to our knowledge, this is the first report of a leaf-colonizing *A. pullulans* strain effectively managing disease in kiwifruit as well as mediating plant defence genes through priming. The ability of CG163 to prime the defence response rather than to cause direct induction of gene expression, like ASM, may explain why repeat applications had no significant effect on ‘Hayward’ plant growth. CG163 also significantly induced the expression of the SA-genes: Class IV Chit, Gluc_PrimerH and PR1_P31 between 5 and 24 h after Psa inoculation. Chitinases are PR proteins, but their target substrate, chitin, a key component in the cell walls of fungal pathogens [36] is not present in a bacterium like Psa. However, chitinases and PR2 proteins often work in tandem to produce greater antimicrobial activity [37]. This study shows the importance of PR2 proteins, while Cellini et al. [15] showed that PR8 chitinase expression was positively associated with Psa control in ‘Hayward’ kiwifruit. Gluc_Primer H has been associated with kiwifruit resistance against Psa [24] and scale insects [38]. PR1_31 is a different isoform of the PR1 protein and may be useful in distinguishing the responses to ASM and CG163. However, CG163 was shown to prime the expression of PR1_P32 when applied one day before Psa inoculation, thus demonstrating the potential importance of application timing.

The additive effect of combining ASM and CG163 was indicated by an increase of β-1,3-glucosidase and PR1 expression compared with that in the treatments applied on their own. β-1,3-glucosidase expression was significantly increased in ASM + CG163 treatments immediately before and at 24 h after inoculation with Psa. Furthermore, ASM + CG163 induced significantly greater PR1 expression at 24 h than either ASM or CG163 alone. The combination treatment downregulated ABA1 expression compared with those in the treatments applied on their own, and in the untreated control. ABA1 itself encodes a zeaxanthin epoxidase that functions in ABA biosynthesis, with its expression enhanced by osmotic stress [39]. ABA is a phytohormone that plays a key role in regulating the plant’s response to biotic and abiotic stresses. Elevation of ABA results in regulation of stomatal aperture and expression of stress-responsive genes. In response to pathogens, the role of ABA is more obscure, although there is increasing evidence supporting its role in regulation of biotic defence responses [40]. The downregulation of ABA1 may be a result of SA accumulation, considering that PR1 and β-1,3-glucosidase operate via the SA-signalling pathways, and the SA pathway is known to be antagonistic with the ABA pathways [41]. Moreover, ASM and CG163 also showed to have significant additive effects on the induction of several other SA-genes when combined. Gluc_PrimerH, Class IV Chit and PR1_P31 were significantly induced between 5 and 48 h after Psa inoculation compared with either component on its own.

Pathogen population studies showed that endophytic Psa populations 96 h after inoculation, were significantly lower on plants treated with ASM + CG163 than in the untreated control. Plants treated with ASM and CG163 also had lower endophytic populations than the control, but the difference was not statistically significant. More detailed time course studies are warranted to gain greater understanding of the complex population dynamics and their relationship with disease expression. These studies should consider spatial factors, in part because Psa has the ability to move systemically trough the plant, and new growth may be susceptible to infection over time. To our knowledge CG163 does not move systemically and so the protection of new growth by ASM + CG163 in treated plants will depend on systemic defence induction. However, induced resistance is typically transient and therefore repeat applications of ASM + CG163 are likely to be required in actively growing plants. The potential risk of growth retardation associated with multiple applications can be offset by preventing the growth penalties that would otherwise result from the disease.

In conclusion, this study demonstrates the additive benefits of combining an elicitor and a BCA to control Psa on kiwifruit. Disease control was greater and was more consistent when ASM and CG163 were combined than when used alone. The activation of host defences by ASM was complemented by the ability of CG163 to directly suppress pathogen populations and to prime host defence genes.

## 4. Materials and Methods

### 4.1. Plant Material

*Actinidia chinensis* var. *deliciosa* ‘Hayward’ tissue cultured plantlets from clonal material (Multiflora, Auckland, New Zealand) were transplanted from ex-flasked sealed punnets with an agar-based growth medium to 0.5 L PB plastic planter bags filled to 2/3 with Daltons™ GB mix (Daltons, Matamata, New Zealand) and topped up with a 50:50 ratio mix of potting mix and perlite. The plants were placed in a glasshouse in high humidity tents with supplementary heating for the first two weeks of growth. Plantlets were further grown in normal glasshouse conditions (15–24 °C, 14 h day length) to approximately 30 cm tall, with 3–4 fully expanded leaves when used for experiments.

### 4.2. Treatments with ASM and A. pullulans CG163 Alone or in Combination

Actigard (Acibenzolar-S-Methyl, Syngenta, Auckland, New Zealand) was prepared in de-ionised water at a concentration of 0.2 g/L and applied as a root treatment (10 mL per plant) or as a spray to just before run-off (spray treatments were only used in the nanostring experiment). *A. pullulans* strain CG163 (CBS Accession #141880) was supplied as a water dispersible granule that was originally sourced from fermentations in 10-L vessels and then formulated into granules at a concentration of 1 × 10^10^ colony forming units (CFU)/g, and stored in a refrigerator at 4 °C until required. CG163 suspensions were prepared in de-ionised water at a concentration of 2 × 10^7^ CFU/mL and applied as a light spray until just before run-off. Treatments were applied seven days before Psa inoculation in the nanostring experiment and seven and one day before pathogen inoculation in the other experiments, unless mentioned otherwise. Spray treatments were performed using a 500-mL hand-operated trigger mist sprayer. All plants were moved into glasshouses immediately after the 2nd treatment application and placed into high-humidity tents (200 × 120 × 82 cm).

### 4.3. Psa3 Inoculum Preparation, Application and Disease Assessment

The Psa culture (strain #10627) was originally isolated in 2010 from an infected *A. chinensis* var. *chinensis* ‘Hort16A’ kiwifruit vine located in the Te Puke region of New Zealand [42]. Psa inoculum was prepared by growing the 10627 strain on King’s B (KB) medium [43] at 28 °C for at least 48 h. The Petri dish culture was washed with sterile de-ionised water to make up a stock solution. The concentration of the bacterial suspension was determined using a NovoSpec™ spectrophotometer (Amersham Biosciences, Auckland, New Zealand) (OD = 600 nm) and then diluted in 10 mM MgSO_4_ with 0.03% (*v*/*v*) wetting agent (Du-Wett^®^), to give a final concentration of c. 2 × 10^7^ CFU/mL. The three youngest fully expanded leaves on each plant were inoculated by placing 6 × 10-µL droplets of Psa inoculum on the underside of each leaf. Plants used for the nanostring experiment were inoculated with 2 × 10^7^ CFU/mL Psa by a CO_2_-pressurized airbrush sprayer on the underside of each leaf, just before run-off. Plants were placed back into (85–100% relative humidity) tents after inoculation with Psa for a week after which the relative humidity was reduced by opening two 20 × 20 cm flaps that had been cut into the top of the plastic tents. Ambient temperature in the glasshouse ranged from 16- to 28 °C. Disease assessment was performed weekly by visually estimating the area of necrotic leaf tissue (mm^2^) at the six original inoculation spots on each leaf. The efficacy of the different treatments was calculated as the percentage reduction in the average area of leaf necrosis relative to that in the untreated controls. For the nanostring experiment, disease severity assessments on each plant were carried out on the leaf immediately above and the leaf immediately below the leaf that had been previously sampled for molecular analysis. Disease severity was determined visually with the aid of a diagram of percentage leaf area infected with Psa and was carried out 10 and 17 days post inoculation.

### 4.4. A. pullulans CG163 Population Analysis

Plants were arranged in three separate groups for population assessment: 2, 7 or 22 days post treatment with CG163. Before treatment, nine immature leaves towards the growing tip and nine fully expanded lower leaves were selected for the population study. All replicates were sprayed with 2 × 10^7^ CFU/mL CG163. For each replicate, five leaf discs were taken from the upper and lower leaf at each time point to determine the population change over time. The leaf disc samples were shaken for 10 min at 130 rpm in 10 mL phosphate buffer 0.05 M + 0.05% Tween^®^ 80 and sonicated for 10 min, on 100% power (Bandelin Sonorex™ digital 10p, Sigma-Aldrich, Auckland, New Zealand). Tenfold serial dilutions were made in sterile de-ionised water to a 1:100 concentration of the original sample. A 100 µL aliquot of the original sample and serial dilutions were plated on Malt Yeast Agar (MYA) plates (10 g malt extract, 1 g yeast extract, and 15 g agar per litre) amended with chloramphenicol (0.1 g/L) and incubated for 48 h at 25 °C. Typical CG163 colonies on the MYA plates were counted and recorded as the number of CFU per cm^2^ of leaf.

### 4.5. Psa3 Population Analysis

Plants were treated as outlined above in ASM and CG163 treatments. A streptomycin-resistant strain of Psa (SR123) was used to facilitate the isolation of Psa. The SR123 strain did not have any fitness and pathogenicity penalties by being streptomycin-resistant. The SR123 population was assessed before inoculation to determine the presence of naturally occurring streptomycin resistant strains on the leaf, just after inoculation (T = 20 min), 48, 72, 96 or 144 h after inoculation. Three fully developed leaves per plant were inoculated with one 10-µL droplet of 2 × 10^7^ CFU/mL Psa, making sure the droplet spread evenly and partially dried before turning the leaf back. At each time point, a total of six leaf discs per treatment were sampled from duplicate plants (one disc per leaf and three leaves per plant). Six discs were also sampled from non-inoculated plants. For the epiphytic population, the discs were placed in 1.5-mL Eppendorf tubes with 1 mL of sterile de-ionised water and vortexed for 15 s to dislodge bacteria from the leaf surface. Samples were ten-fold serially diluted to a 1:10,000 dilution of the original sample and 10-µL droplets of each dilution and original sample were pipetted in triplicate onto KB plates amended with streptomycin (0.1 g/L) and cycloheximide (0.1 g/L). For the assessment of endophytic populations, the same leaf discs were then triple-surface sterilized (3 min 1% *v*/*v* sodium hypochlorite, rinsed with sterile de-ionised water (SDW), followed by 3 min 70% *v*/*v* ethanol soak, and then rinsed with SDW, 3 min 1% *v*/*v* sodium hypochlorite and three SDW rinses). A sub-sample of the final wash solution (10-µL aliquots) was pipetted in triplicate onto KB plates to ensure that all epiphytes had been killed. The leaf samples were homogenised for 40 s on 6 m/s (MP Fastprep^®^ -24) with 300 µL SDW in 2 mL freestanding micro-lysis tubes (Labcon, Petaluma, CA, USA) with five ceramic beads. Leaf samples were serial diluted and plated as described for the epiphytic population assessment. Plates were incubated for at least 48 h at 28°C. Colonies were counted and expressed as CFU/cm^2^ of leaf. Endophytic populations were counted only if no bacteria were present in the final wash.

### 4.6. Plant Growth Analysis

Plant growth was assessed by weekly measurements of stem length (cm) from the bottom of the stem (at the soil level) to the top of the stem at the point of the growing tip. Fifteen 30 to 50 cm high plants per treatment used for this experiment were treated with weekly treatments of ASM (0.2 g/L root treatment), CG163 2 × 10^7^ CFU/mL (spray treatment), a combination of ASM 0.2 g/L (root treatment) and CG163 2 × 10^7^ CFU/mL (spray treatment) or an untreated control.

### 4.7. RNA Extraction

Treated/untreated leaves were sampled just before inoculation, 5, 24, 48 or 96 h after inoculation with Psa and were snap frozen in liquid nitrogen, followed by storage at −80 °C until RNA extraction. Leaf discs were sampled from the inoculation site using a cork borer (18 mm diameter) for the quantitative polymerase chain reaction (qPCR) experiment and whole leaves with the mid vein of each sampled leaf removed were sampled for the nanostring experiment. Total RNA from 100 mg samples of pulverised leaf material was extracted using the Spectrum™ plant total RNA kit (Sigma-Aldrich, Auckland, New Zealand) according to the manufacturer’s instructions. RNA extracted from the leaf tissue was treated with a DNA removal kit Quanta PerfeCTa DNase I kit (DNature, Gisborne, New Zealand), to remove any contaminant genomic DNA (gDNA). DNAse-treated RNA samples were checked by PCR to confirm that there was no gDNA contamination. cDNA synthesis, using 1-µg RNA per single positive reverse transcriptase reaction for each sample, was performed according to manufacturer’s instructions using Quanta qScript cDNA Supermix kit (DNature, Gisborne, New Zealand). 

### 4.8. Gene Expression Analysis by Real-Time Quantitative PCR

Quantitative PCR (qPCR) was performed in triplicate on the samples, in a 10-µL reaction volume containing 1-µL of cDNA (diluted 25-fold in nuclease-free water), 1-µL each of forward and reverse primers (10-µM), and 5-µL of Light Cycler^®^ 480 SYBR Green 1 Master Mix (Roche Diagnostics GmbH, Mannheim, Germany). Selection of reference genes (RGs) was based on stability of expression in other qPCR studies on kiwifruit [44,45]. After testing eight different RGs, the two RGs that were most stably expressed under the conditions of each experiment as determined using GeNorm software (40 s ribosomal protein and protein phosphatase 2A) were used for normalization. Primers for RGs and genes of interest (GoI) were designed using Primer3 software (The Whitehead institute, Cambridge, MA, USA) and were synthesised by Invitrogen (Auckland, New Zealand), except for pathogenesis related protein 1 (PR1) [15] and protein phosphatase [45]. A gene expression normalization factor (N), calculated using geNorm software v3.4 [46] for each sample, based on the geometric mean of the RGs, was used for the calculation of relative expression of each GoI. The basal transcript level in untreated plants (Baseline treatment) for 0 h and 24 h after treatments was then used as a further reference point for all calculations and is referred to with the value 1. The relative quantification thermal cycling conditions were: denaturation at 95 °C for 10 min, followed by 40 cycles of 15 s denaturation at 95 °C, 15 s annealing at a different optimised temperature between 55 and 60 °C for each primer set, and 20 s extension at 72 °C. Inter-run variability was controlled by including a complete set of treatments on each plate, but a separate run for each biological replicate (i.e., three runs/primer set, which were then averaged). Melting curve analysis (60–95 °C at 1 °C increments with 5 s between each step) was performed after the final qPCR cycle to validate amplicon specificity. Non-template controls were also included to assess the purity of the reagents.

### 4.9. Gene Expression Analysis by CodeSet^®^ Nanostring

Samples comprising one pre-marked ‘Hayward’ leaf per plant per replicate were collected 5, 24, 48 and 96 h after inoculation with Psa, with three replicates/treatment and two plants per replicate. Total RNA from 100 mg samples of pulverised leaf material was extracted as described for the RNA extraction. RNA concentration and purity were assessed by measurement of 1 µL of sample mixed with 4 µL RNAse free water on a NanoDrop™ 200c spectrophotometer (Thermo Scientific, Waltham, MA, USA). The minimal acceptable concentration was 100 ng purified intact total RNA at ≥20 ng/μL. The samples (25 μL of each sample with a concentration averaging 200 ng/μL) were sent on dry ice to Otago Genomics (Dunedin, New Zealand) performing CodeSet Nanostring, where RNA quantity and quality were further checked by Agilent 2100 BioAnalyser (ThermoFisher Scientific, Waltham, MA, USA) and Qubit (ThermoFisher Scientific, Waltham, MA, USA). Samples were then stored at −80 °C until the time of analysis.

Selection of RGs used for CodeSet Nanostring are described in the gene expression analysis by real time quantitative PCR (see above). GoI were initially chosen to represent as many different defence response pathways and temporal stages of defence as possible. Details of the genes are found in Supplementary Table S1.

For each sample, 100 ng total RNA in 5 μL total volume was processed by using the standard nCounter gene expression total RNA protocol (NTI). In brief, the total RNA and CodeSet Probes (designed and synthesized by NTI) were combined with hybridization buffer (NTI) and incubated at 65 °C for 22 h. The CodeSet Probes consisted of a reporter and a capture probe of 55 gene targets (including seven reference genes) that hybridize to the target sequences of interest, forming a tripartite complex. Hybridized samples were then processed in batches of 12 by using a high sensitivity protocol for 3 h for each 12 sample cartridge on the robotic prep station (NTI). Data acquisition was performed by using the GEN2 Digital Analyzer (NTI), with the “max” field of view setting (555 images per sample; 5 h scan per cartridge). Raw data generated by the GEN2 Digital Analyzer were exported as RCC files and quality control (QC) checked by using nSolver™ software (NTI). All data passed QC and were further analysed with nSolver™ software at PFR. nSolver software was used to check: (1) counts associated with positive and negative controls (present in all tubes) to ensure that nanostring was amplifying correctly, without too much noise; (2) that the RGs were providing stable expression (checked by geNORM analysis), and; (3) that none of the data raised any QC flags (data not shown, as no QC flags were present). Gene expression data were then normalised against all the RGs; the mixed sample, which was run across all eight PlexSets; and against the control (water treatment) at each equivalent time point. Data are presented in tables, in which the relative decrease/increase in expression of the GoIs in treatments relative to the control at each different time point. The relative value of 1 represents an unchanged value; when A/B is less than 1, the fold change displays as the negative reciprocal. A change of ≥2 fold is generally used as a bioinformatics cut-off [47,48,49]. 

### 4.10. Statistical Analysis

Data for disease severity, population studies and growth experiments were subjected to a general analysis of variance (ANOVA) and if required, were transformed to satisfy the assumptions of ANOVA (normal distribution and homogeneity of variances). Data from bacterial population counts were fitted using generalized linear mixed model with Poisson errors (link = log), time and treatment were fitted as fixed effects; leaves nested within plants were fitted as random effects. Post-hoc differences in bacterial population between treatments at each time point were determined by 95% confidence intervals (CIs). Non-overlapping CIs indicated significant difference. Data were analysed using Genstat 19th edition for Windows. Generalized linear mixed model (GLMM) and Fisher’s Least Significant Difference (LSD) means comparison tests were applied with a 5% significance level.

## Figures and Tables

**Table 1:** Expression of Class IV chitinase (ClassIV_Chit), downy mildew resistant 6 (DRM6), benzyl alcohol dehydrogenase (BAD), pathogenesis-related protein 1 (PR1_P31), pathogenesis-related protein 1 (PR1_P32), thaumatin-like protein (TLP_TG4) and glucan endo 1,3-β-glucosidase (Gluc_PrimerH) genes in *Actinidia chinensis* var. *deliciosa* ‘Hayward’ kiwifruit treated with a single foliar spray of acibenzolar-S-methyl (ASM), *Aureobasidium pullulans* isolate CG163 (CG163), combination of ASM and CG163 (ASM + CG163) or untreated (control). This was followed by spray inoculation with 2 × 10^7^ CFU/mL *Pseudomonas syringae* pv. *actinidiae* 7 days after treatment, and sampling of leaf tissue 5, 24, 48 and 96 h after inoculation. Gene expression was measured by CodeSet^®^ Nanostring and was normalised against expression of seven reference genes (see Supplementary Table S1 for details). Values are means of three independent biological replicates and are based on fitted mean back-transformed data. Gene-expression between different treatments and time points are compared within each separate column/gene. Treatments with different letters are significantly different according to Fisher’s Least Significant Difference test (LSD) at the 5% level. Significant increases in gene expression compared with those in the untreated control are highlighted in bold. Asterisks indicate statistically significant differences in gene expression between ASM + CG163 treatments and a single treatment of either ASM or CG163.

Time	Treatment	Class IV Chit	DRM6	BAD	PR1_P31	PR1_P32	TLP_TG4	Gluc_PrimerH
5 h	Control	1011 ab	794 b	41 a	10 a	2415 b	293 ab	691 ab
	ASM	1085 ab	**3733** f	**398** e	13 ab	**16,260** d	**2854** fg	822 abc
	CG163	**1684** cd	445 a	43 a	**40** cde	1339 ab	248 a	**1561** cdef
	ASM + CG163	2922 * e	**3146** ef	**167** c	**69** defg	**19,305** d	**3233** fg	3472 * g
24 h	Control	963 a	2870 e	73 b	18 abc	1098 a	565 bc	513 a
	ASM	**1514** bc	**9985** g	**319** de	30 bcd	**21,076** d	**8145** hi	1016 abcd
	CG163	**1918** cd	2489 de	83 b	35 bcd	1826 ab	824 cd	**1573** cdef
	ASM + CG163	3721 * ef	**9175** g	**225** cd	131 * fg	**22,615** d	**13,044** i	**2282** efg
48 h	Control	1804 cd	1771 c	66 ab	100 efg	1501 ab	1193 cde	1315 bcde
	ASM	1861 cd	**9060** g	**167** c	51 def	**20,919** d	**5731** gh	884 abc
	CG163	2526 de	1774 c	67 ab	39 cde	1636 ab	846 cde	1526 cdef
	ASM + CG163	5650 * g	**8464** g	**144** c	150 gh	**23,906** d	**12,102** hi	**2833** fg
96 h	Control	7208 g	1988 cd	210 cd	5839 k	5600 c	1839 ef	1534 cdef
	ASM	5537 fg	**9942** g	226 cd	1376 j	**25,479** d	**13,230** i	2007 defg
	CG163	3645 e	1953 cd	145 c	698 ij	2453 b	1592 def	1348 bcdef
	ASM + CG163	7002 g	**9225** g	189 c	399 hi	**23,423** d	**15,536** i	2155 defg

**Table 2:** Expression of lipoxygenase 2 (LOX2), APETALA2 ethylene responsive factor 2 (AP2_ERF2), respiratory burst oxidase homolog gene F (RBOHF) and oxidative stress 2 zinc finger (OXS2_Zn_finger) genes in *Actinidia chinensis* var. *deliciosa* ‘Hayward’ kiwifruit treated with a single foliar spray of acibenzolar-S-methyl (ASM), *Aureobasidium pullulans* isolate CG163 (CG163), combination of ASM and CG163 (ASM + CG163) or untreated (control). This was followed by spray inoculation with 2 × 10^7^ CFU/mL *Pseudomonas syringae* pv. *actinidiae* 7 days after treatment, and sampling of leaf tissue 5, 24, 48 and 96 h after inoculation. Gene expression was measured by CodeSet Nanostring and was normalised against expression of seven reference genes (see Supplementary Table S1 for details). Values are means of three independent biological replicates and are based on fitted mean back-transformed data. Gene-expression between different treatments and time points are compared within each separate column/gene. Treatments with different letters are significantly different according to Fisher’s Least Significant Difference test (LSD) at the 5% level. Significant increases in gene expression compared with those in the untreated control are highlighted in bold. Asterisks indicate statistically significant differences in gene expression between ASM + CG163 treatments and a single treatment of either ASM or CG163.

Time	Treatment	LOX2	AP2_ERF2	RBOHF	OXS2_Zn_Finger
5 h	Control	925 bcde	24 ab	167 a	180 bc
	ASM	1244 def	29 abc	**327** bc	**260** defg
	CG163	920 bcde	27 abc	158 a	170 b
	ASM + CG163	1645 efg	48 abcd	214 ab	208 bcde
24 h	Control	476 a	16 a	375 cd	277 efg
	ASM	711 abcd	19 a	581 de	**448** hi
	CG163	666 abc	16 a	354 c	341 fgh
	ASM + CG163	**1214** def	**73** bcd	589 de	**440** hi
48 h	Control	674 abc	90 cd	299 bc	170 b
	ASM	630 ab	17 a	**816** e	**402** hi
	CG163	542 ab	19 a	390 cd	**250** cdef
	ASM + CG163	1176 cdef	47 abcd	**801** e	**344** fgh
96 h	Control	6983 h	2525 g	293 bc	113 a
	ASM	2515 g	628 f	635 e	**364** gh
	CG163	1925 fg	458 ef	362 c	**279** efg
	ASM + CG163	1685 fg	128 de	1299 * f	**522** i

## Supplementary Materials

The following are available online at https://www.mdpi.com/2223-7747/8/8/287/s1, Table S1: Identification of 7 reference genes (RGs) and 12 genes of interest (GoI) used for gene expression analysis of plant defence responses by CodeSet™ Nanostring Table S2: Log10 population data of three separate experiments, with time-points of 48 and 96 h (Experiment 1), 48 and 144 h (Experiment 2) and 72 and 144 h (Experiment 3), measuring average epiphytic and endophytic populations of *Pseudomonas syringae* pv. *actinidiae* (CFU/cm^2^ of leaf) in *Actinidia chinensis* var. *deliciosa* ‘Hayward’ potted plants. Plants were treated with acibenzolar-S-methyl (ASM), *Aureobasidium pullulans* isolate CG163 (CG163), combination of ASM and CG163 (ASM + CG163) or were left as an untreated control 7 and 1 day before inoculation with *Pseudomonas syringae* pv. *actinidiae*. Values are means of two biological replicates for Experiments 1 + 2 and six biological replicates for Experiment 3. Each biological replicate consisted of an average of one inoculation spot on three (Experiment 1 + 2) or two (Experiment 3) different leaves on the same plant. Bacterial populations just after inoculation consisted of two (Experiments 1 + 2) or six (Experiment 3) untreated biological replicates for all treatments, with each biological replicate consisting of an average of one inoculation spot on three (Experiments 1 + 2) or two (Experiment 3) different leaves on the same plant. Log10 values of epiphytic and endophytic bacterial populations with different letters (highlighted in bold) signifying differences according to average back-transformed bacterial populations of a generalized linear mixed model (GLMM) with a 95% confidence interval.
