# Supplementary material for: Integrated Use of Aureobasidium pullulans Strain CG163 and Acibenzolar-S-Methyl for Management of Bacterial Canker in Kiwifruit
Source: Plants (Basel). 2019 Aug 15;8(8):287. doi: 10.3390/plants8080287 (PMC6724088; doi:10.3390/plants8080287)
Supplement: Supplementary file 1 [file plants-08-00287-s001.pdf]

Table S1: Identification of 7 reference genes (RGs) and 12 genes of interest (GoI) used for gene expression analysis of plant defence responses by CodeSet™ Nanostring

Gene name (Gene ID used in Figures)	Reference Gene (RG) or Gene of Interest (GoI)	Equivalent Red5 Acc. number[1]	GeneBank ID or Achn number[2]	Reason for selection & most relevant references
Elongation Factor (EF)	RG	Acc31629.1	FG526520	Stable RG in kiwifruit/Psa interactions [24,45]
40s ribosomal protein (40s)	RG	Acc23370.1	FG498176	Stable RG in kiwifruit/Psa interactions [24,50].
Beta tubulin (β-tub)	RG	Acc23370.1	BAB10059/FG523755	Stable RG in kiwifruit/Psa interactions [45].
Ubiquitin-conjugating enzyme (UBC)	RG	Acc01363.1	FG478277	Stable RG in kiwifruit/Psa interactions [45].
Glyceraldehyde 3-phosphate dehydrogenase (GAPDH)	RG	Acc18050.1	AAF78494/FG499278	Stable RG in kiwifruit/Psa interactions [45].
Protein phosphatase 2A (PP2A)	RG	Acc33246.1	FG522516	Stable RG in kiwifruit/Psa interactions [45].
Actin (Actin2)	RG	Acc05529.1	EF063572	Stable RG in kiwifruit/Psa interactions [15,38,45,50].
Pathogenesis-related protein family 1 (PR1_P31)	GoI	Acc07445.1	Achn240741	PR1 is an SA marker. This isoform shows some ability to distinguish between Psa and *Pseudomonas syringae* pv. *actinidifoliorum* (Pfm) [38].
Pathogenesis-related protein family 1 (PR1_P32)	GoI	Acc06864.1	FG499230	PR1 is the most commonly used marker of SA responses, one of the best qPCR markers in the laboratory and the field [15,38].
Lipoxygenase 2 (LOX2)	GoI	Acc32487.1	Achn123621	Commonly used marker of the JA pathway [51,52].
APETALA2 Ethylene responsive factor 2 (AP2_ERF2)	GoI	Acc20680.1	Achn033321	Ethylene responsive Transcription factor in the JA pathway – JA and ethylene interact to activate plant defensins [53]. Involved in kiwifruit/Psa interaction [38].

Abscisic acid deficient 1 (ABA1)	GoI	Acc10322.1	Achn013171	Involved in abscisic acid (ABA) biosynthesis. Commonly used marker of the ABA pathway [54,55].
Glucan endo-1,3-β-glucosidase (Gluc_PrimerH)	GoI	Acc03929.1	FG455092	Thought to convert preformed inert phytoanticipins (synthesized via the PPP) into their corresponding toxic aglycones [25]. Involved in kiwifruit/Psa interaction [38].
Thaumatin-like protein (TLP_TG4)	GoI	Acc28854.1	AJ871175	PR5 protein involved in resistance to *Botrytis cinerea*, to which it is directly antifungal [56,57], and ripe rot of kiwifruit [38,44].
Class IV chitinase (ClassIV_Chit)	GoI	Acc00338.1	FG457667	PR3 correlated with elicitor-induced defence against *Cryptosporiopsis* [44,58].
Benzyl alcohol dehydrogenase (BAD)	GoI	Acc01173.1	Achn239251	One of the most differentially expressed genes a Psa-inoculated kiwifruit cane next generation sequencing experiment (A. Allan, PFR, unpublished).
Downy mildew resistant 6 (DMR6)	GoI	Acc03241.1	Achn045721	One of the most differentially expressed genes a Psa-inoculated kiwifruit cane next generation sequencing experiment (A. Allan, PFR, unpublished). Possible modulator of the SA response [28].
Oxidative stress 2 zinc finger (OXS2_Zn_finger)	GoI	Acc15109.1	Achn128681	One of the most differentially expressed genes a Psa-inoculated kiwifruit cane next generation sequencing experiment (A. Allan, PFR, unpublished). TF involved in activating stress tolerance [59].
Respiratory burst oxidase homolog gene F (RBOHF)	GoI	Acc15636.1	Achn052291 (worse match than RBOHA)	One of the most differentially expressed genes in a Psa-inoculated kiwifruit cane next generation sequencing experiment (A. Allan, PFR, unpublished). Allows for fine tuning of reactive oxygen species (ROS) production in response to biotic and abiotic stresses [60].

[1] Acc. numbers come from the manual annotation of the Red5 kiwifruit whole Genome Shotgun project, that has been deposited at DDBJ/ENA/GenBank under the accession NKQK00000000 and is discussed by Pilkington, S. et al. (2018) BMC Genomics 19:257.

[2] *A. chinensis* var. chinensis 'Honyang' genome sequences are from the project described by Huang et al. in Nature Communications doi:10.1038/ncomms3640. The sequences are found at http://bioinfo.bti.cornell.edu/cgi-bin/kiwi/home.cgi Type in the http address, select search, then type in the Achn number.

References

50. Wurms, K.; Gould, E.; Chee, A.A.; Taylor, J.; Curran, B.; Reglinski, T. Elicitor induction of defence genes and reduction of bacterial canker in kiwifruit. *N. Z. Plant Prot.* **2017**, *70*, 272–284.
51. Garcia-Marcos, A.; Pacheco, R.; Manzano, A.; Aguilar, E.; Tenllado, F. Oxylipin biosynthesis genes positively regulate programmed cell death during compatible infections with the synergistic pair potato virus x-potato virus y and tomato spotted wilt virus. *J. Virol.* **2013**, *87*, 5769–5783.
52. Wasternack, C.; Hause, B. Jasmonates: Biosynthesis, perception, signal transduction and action in plant stress response, growth and development. An update to the 2007 review in annals of botany. *Ann. Bot.* **2013**, *111*, 1021–1058.
53. Pré, M.; Atallah, M.; Champion, A.; De Vos, M.; Pieterse, C.M.J.; Memelink, J. The ap2/erf domain transcription factor ora59 integrates jasmonic acid and ethylene signals in plant defense. *Plant Physiol.* **2008**, *147*, 1347–1357.
54. Ding, Z.H.; Li, S.M.; An, X.L.; Liu, X.J.; Qin, H.M.; Wang, D. Transgenic expression of myb15 confers enhanced sensitivity to abscisic acid and improved drought tolerance in *arabidopsis thaliana*. *J. Genet. Genomics* **2009**, *36*, 17–29.
55. Sanchez-Vallet, A.; Lopez, G.; Ramos, B.; Delgado-Cerezo, M.; Riviere, M.P.; Llorente, F.; Fernandez, P.V.; Miedes, E.; Estevez, J.M.; Grant, M., *et al.* Disruption of abscisic acid signaling constitutively activates *arabidopsis* resistance to the necrotrophic fungus *plectosphaerella cucumerina*. *Plant Physiol.* **2012**, *160*, 2109–2124.
56. Wang, H.X.; Ng, T.B. Isolation of an antifungal thaumatin-like protein from kiwi fruits. *Phytochemistry* **2002**, *61*, 1–6.
57. Wurms, K.; Greenwood, D.; Sharrock, K.; Long, P. Thaumatin-like protein in kiwifruit. *J. Sci. Food Agric.* **1999**, *79*, 1448–1452.
58. Wurms, K.; Cui, W.; Ah Chee, A.; Rees-George, J.; Bublin, M.; Breiteneder, H. Down regulation of putative defence-associated transcripts correlates with ripe rot symptoms on kiwifruit (*actinidia chinensis*). *J. Phytopathol.* **2011**, *159*, 435–442.
59. Blanvillain, R.; Wei, S.; Wei, P.C.; Kim, J.H.; Ow, D.W. Stress tolerance to stress escape in plants: Role of the oxs2 zinc-finger transcription factor family. *Embo J.* **2011**, *30*, 3812–3822.
60. Han, J.P.; Koster, P.; Drerup, M.M.; Scholz, M.; Li, S.Z.; Edel, K.H.; Hashimoto, K.; Kuchitsu, K.; Hippler, M.; Kudla, J. Fine-tuning of rbohf activity is achieved by differential phosphorylation and ca2+ binding. *New Phytol.* **2019**, *221*, 1935–1949.

Table S2: Log10 population data of three separate experiments, with time-points of 48 and 96 h (experiment 1), 48 and 144 h (experiment 2) and 72 and 144 h (experiment 3), measuring average **e**piphytic and endophytic populations of *Pseudomonas syringae* pv. *actinidiae* (CFU/cm^2 of leaf) in *Actinidia chinensis* var. *deliciosa* 'Hayward' potted plants. Plants were treated with acibenzolar-S-methyl (ASM), *Aureobasidium pullulans* isolate CG163 (CG163), combination of ASM and CG163 (ASM+CG163) or were left as an untreated control 7 and 1 day before inoculation with *Pseudomonas syringae* pv. *actinidiae*. Values are means of two biological replicates for experiments 1+2 and six biological replicates for experiment 3. Each biological replicate consisted of an average of one inoculation spot on three (experiment 1+2) or two (experiment 3) different leaves on the same plant. Bacterial populations just after inoculation consisted of two (experiments 1+2) or six (experiment 3) untreated biological replicates for all treatments, with each biological replicate consisting of an average of one inoculation spot on three (experiments 1+2) or two (experiment 3) different leaves on the same plant. Log10 values of epiphytic and endophytic bacterial populations with different letters (highlighted in bold) signifying differences according to average back-transformed bacterial populations of a generalized linear mixed model (GLMM) with a 95% confidence interval.

Exp.	Time	Treatment	Epiphytic population (Log10)	Endophytic population (Log10)
**1.**	48 h	Control	5.54 a	4.96 a
		ASM	4.67 ab	3.92 a
		CG163	**4.24 b**	2.81 a
		ASM+CG163	4.42 ab	3.82 a
	96 h	Control	6.18 a	6.08 a
		ASM	6.01 ab	4.27 a
		CG163	**3.92 b**	5.37 a
		ASM+CG163	4.68 ab	3.38 a
**2.**	48 h	Control	4.53 a	4.92 a
		ASM	4.35 a	4.52 a
		CG163	3.84 a	5 a
		ASM+CG163	4 a	4.57 a
	144 h	Control	7.33 a	6.37 a
		ASM	6.32 ab	5.85 a
		CG163	**4.3 b**	5.36 ab
		ASM+CG163	**4.54 b**	**3.84 b**
**3.**	72 h	Control	6.25 a	5.27 a
		ASM	6.11 a	4.83 a
		CG163	**5.19 b**	5.25 a
		ASM+CG163	5.51 ab	4.05 a
	144 h	Control	5.92 a	6.30 a
		ASM	6.13 a	5.49 ab
		CG163	5.69 a	5.21 ab
		ASM+CG163	5.24 a	**3.1 b**
